# Complete genome sequence of *Sphingobium yanoikuyae* strain K1B isolated from the pea rhizosphere

**DOI:** 10.1128/mra.00470-26

**Published:** 2026-06-25

**Authors:** P. V. Guro, E. A. Sekste, T. S. Azarova, A. I. Shaposhnikov, O. S. Yuzikhin, V. Y. Shahnazarova, N. A. Vishnevskaya, M. I. Lebedinskii, V. I. Safronova, A. A. Belimov, A. L. Sazanova

**Affiliations:** 1 All-Russia Research Institute for Agricultural Microbiology (ARRIAM) 117473 , St. Petersburg, Russia; DOE Joint Genome Institute, Berkeley, California, USA

**Keywords:** bacteria, genome sequence, soil, plant rhizosphere

## Abstract

We report the genome sequence of *Sphingobium yanoikuyae* strain K1B isolated from the pea rhizosphere. The genome consists of a 4.80 Mb chromosome and three plasmids of 285 kb, 177 kb, and 39 kb, and contains 4,937 protein-coding genes and 77 RNA genes.

## ANNOUNCEMENT

Bacteria from the genus *Sphingobium* are common inhabitants of soil and the plant rhizosphere, where they contribute to the degradation of aromatic compounds and mediate various interactions between plants and microorganisms (1). Members of this genus have gained attention as plant growth-promoting rhizobacteria (PGPR) due to their ability to produce the phytohormone indole-3-acetic acid (IAA), extract iron through siderophores, and improve nutrient acquisition in plants (2, 3). Strain K1B was isolated in 2024 from the rhizosphere of pea (*Pisum sativum* L.) in Veliky Novgorod region, Russia (57.148,279°N, 31.179,109°E). An original growth medium supplemented with abscisic acid (ABA) as the sole carbon source was used for isolating the strain, obtaining pure culture, and preparing separate colonies for further study (4). Colonies appeared after 3 days at 28°C. A single colony was re-isolated twice. DNA was extracted using DNeasy Blood & Tissue Kit (Qiagen) from culture grown in broth at 28°C for 48 h, with shaking at 180 rpm. A sequencing library was prepared with the Ligation Sequencing gDNA - Native Barcoding Kit 24 v14 (ONT) without fragmentation. Sequencing was performed on a MinION R10 flow cell (ONT). Basecalling used Dorado v0.7.3. Quality control of raw reads was performed with NanoStat v1.6.0 (5). Sequencing produced 40,000 reads (average length 8,477.3 bp, N50 17,687.0 bp, mean quality 12.8). Reads were filtered with Filtlong v0.2.1 (--min_length 1000; --keep_percent 95), removing short and low-quality reads while retaining 95% of total bases, resulting in 27,838 reads with an average length of 11,572 bp. The genome sequence was obtained by consensus long-read assembly using Trycycler v0.5.4 (6). Filtered reads were subsampled into 12 subsets. Each was assembled with Flye v2.9, Miniasm v0.3 with Minipolish v0.1.3, and Raven v1.8.1 (7–10). Assemblies were clustered into four replicons by Trycycler cluster. For each cluster, multiple alignment used Trycycler msa (Muscle v3.8.31), with reads partitioned via Trycycler partition (11). Final consensus sequences were generated with Trycycler consensus and polished with Medaka v1.8.0 (r1041_e82_400bps_sup_v4.2.0). Genome statistics were measured using QUAST v5.2.0 (12). The total size of the assembly is 5.30 Mbp distributed across four circular replicons: a main chromosome of 4,797,990 bp and three plasmids of 285,295, 176,986, and 39,218 bp. The N50 value is 4,797,990 bp, with an average coverage of 62.2× and a GC content of 64.5%. Default parameters were used for all software unless otherwise noted. Annotation by NCBI PGAP v6.10 identified 5,143 total genes: 4,937 protein-coding, 77 RNA genes (12 rRNAs, 61 tRNAs, 4 ncRNAs) (13). For taxonomic identification, average nucleotide identity (ANI) was calculated using FastANI v1.33 and digital DNA-DNA hybridization (dDDH) using the Type Genome Server (14, 15). Comparison with type strains revealed 96.69% ANI and 70.4% dDDH with *S. yanoikuyae* HAMBI 1842 (GCA_034424525.1), confirming strain K1B as *Sphingobium yanoikuyae*.

## Data Availability

All data are available at GenBank under BioProject accession number PRJNA1424895. The BioSample accession number is SAMN55393615. The raw MinION data can be found under number SRR37260526, and the assembly accession numbers are CM144252.1 (chromosome) and JBVDQO010000002.1–JBVDQO010000004.1 (plasmids).
